# Epigenetic mechanisms of drug resistance in acute myeloid leukemia: advances in small-molecule targeted therapy

**DOI:** 10.3389/fphar.2025.1672524

**Published:** 2025-10-20

**Authors:** Jialing Tang, Xibo Ye, Zhangyang Xu, Tianjiao Min, Zherun Tian, Li Ma, Ying He, Beier Jiang

**Affiliations:** ^1^ Naval Medical Center of PLA, Shanghai, China; ^2^ State Key Laboratory of Eye Health, Department of Ophthalmology, Shanghai Ninth People’s Hospital, Shanghai Jiao Tong University School of Medicine, Shanghai, China

**Keywords:** acute myeloid leukemia, drug resistance, epigenetic dysregulation, combination strategies, relapse

## Abstract

Acute myeloid leukemia (AML) remains a formidable therapeutic challenge, with high relapse rates rooted not only in genetic heterogeneity, but also in dynamic and reversible epigenetic dysregulation that drive resistance to conventional and targeted small-molecule therapies. Although small-molecule inhibitors targeting epigenetic regulators have demonstrated preclinical efficacy and initial clinical activity, therapeutic gains are often limited by transient responses, intra- and inter-patient heterogeneity, and an incomplete understanding of predictive biomarkers. Besides, clinical outcomes have often been less robust than preclinical data suggested, partly due to the complexity of AMLs genetic and epigenetic heterogeneity, the limitations of model systems, and intrinsic resistance mechanisms. Combination approaches, such as pairing epigenetic agents with chemotherapy, immunotherapy, or other targeted drugs are under investigation. However, clinical efficacy remains inconsistent and requires a mechanistic rationale to avoid antagonism. To move forward, it is essential to delineate specific epigenetic dependencies in resistance-prone AML subtypes, design rational, biomarker-guided combination regimens, and enhance specificity and pharmacodynamic profiles of epigenetic inhibitors. Such an integrated strategy holds promise for elevating the effectiveness of epigenetic therapies in AML. Continued research is essential to refine epigenetic-based precision medicine and ultimately improve patient outcomes.

## 1 Introduction

AML is a highly aggressive hematologic malignancy characterized by substantial biological heterogeneity, driven by the clonal expansion of hematopoietic stem and progenitor cells. It is the most common form of acute leukemia in adults, with a median age at diagnosis of approximately 68 years, and its incidence increases with age. The 5-year-survival rate of patients diagnosed with AML before 10 years ago was only 29% (below 30%) when utilizing conventional chemotherapy (Forsberg and Konopleva, 2024). Notably, AML is responsible for the highest proportion of leukemia-related mortality among all leukemia subtypes, particularly pronounced in older adults (Shallis et al., 2019; Sasaki et al., 2021). Clinically, AML presents with a variety of symptoms, including anemia, infections, bleeding, bone and joint pain, central nervous system involvement, and signs related to leukemic cell infiltration. Additionally, AML can also lead to severe complications such as hemorrhage, bone marrow failure, increased susceptibility to infections, and potentially life-threatening outcomes (Dohner et al., 2010).

For several decades, the traditional chemotherapy for AML has been the “7 + 3” regimen, consisting of a 3-day course of daunorubicin combined with a 7-day course of cytarabine (Kantarjian et al., 2021). Despite its widespread use, clinical outcomes remain suboptimal. A large retrospective study of patients under the age of 55 revealed 5-year overall survival (OS) rates of 64%, 41%, and 11% for favorable, intermediate, and adverse risk groups, respectively. Although approximately 40% of patients achieve complete remission (CR), the median OS remains limited to 12–18 months (Pelcovits and Niroula, 2020). These unsatisfactory outcomes are primarily attributed to the significant genetic and molecular heterogeneity of AML, which contributes to therapeutic resistance and disease relapse (Do and Byrd, 2015).

Therapeutic resistance remains one of the most formidable barriers to curative treatment in AML. While many patients initially respond to induction chemotherapy, a substantial proportion eventually experience relapse, often with a more aggressive disease phenotype and reduced responsiveness to subsequent treatments. This resistance is driven by a complex network of biological mechanisms, including mutations in key regulatory genes (e.g., *FLT3, TP53, NPM1*), overexpression of drug efflux transporters (e.g., P-glycoprotein), evasion of apoptosis, and metabolic reprogramming that enables leukemic cells to survive chemotherapy-induced stress. Furthermore, leukemic stem cells (LSCs), which are inherently resistant to conventional therapies, persist after treatment and contribute to long-term disease maintenance. Interactions between leukemic cells and the bone marrow microenvironment reinforce resistance by creating a protective niche that promotes cell survival and diminishes drug efficacy. Notably, emerging evidence highlights the critical role of epigenetic dysregulation in AML chemoresistance, suggesting a potential avenue for therapeutic intervention (Esposito and So, 2014; Manivannan et al., 2025).

Among the diverse mechanisms contributing to therapeutic resistance in AML, epigenetic dysregulation has emerged as a central driver of treatment failure (Yu et al., 2025). Mutations in key epigenetic regulators such as DNA (cytosine-5)-methyltransferase 3 alpha (DNMT3A), ten-eleven translocation 2 (TET2), and isocitrate dehydrogenase 1/2 (IDH1/2) lead to aberrant DNA methylation profiles that impair hematopoietic differentiation and pre-chemoresistance (Gurnari et al., 2020; Mishra et al., 2023a). Dysregulated activity of chromatin-modifying enzymes, including enhancer of zeste homolog 2 (EZH2) and histone deacetylases (HDACs), represses the expression of pro-apoptotic genes, thereby enhancing leukemic cell survival during treatment (Mishra et al., 2023b). Epigenetic abnormalities also facilitate leukemogenesis and support the persistence of quiescent, drug-resistant LSCs, which are a major cause of relapse. Notably, unlike genetic mutations, epigenetic changes are potentially reversible, offering promising opportunities for therapeutic intervention. Pharmacologic targeting of epigenetic regulators has been shown to restore normal gene expression and sensitize AML cells, including LSCs, to both conventional and novel therapies (Esposito and So, 2014; Manivannan et al., 2025).

Given the critical role of epigenetic dysregulation in AML drug resistance, the exploration of epigenetic mechanisms underlying drug resistance and the development of small-molecule targeted therapies have become key areas of cancer research, especially for hematologic tumors. Small-molecule inhibitors targeting specific epigenetic pathways hold great potential for reversing drug resistance in AML cells and enhancing therapeutic efficacy (Issa et al., 2021). In this review, we summarize the major epigenetic mechanisms contributing to drug resistance in AML and provide a comprehensive overview of recent advances in small-molecule targeted therapies aimed at reversing epigenetic-driven resistance. Furthermore, epigenetic abnormalities present a strong mechanistic basis for the development of combination therapies. By strategically integrating agents with distinct mechanisms of action, it is possible to enhance treatment tolerability, reduce toxicity, and increase therapeutic efficacy—considerations that are particularly important for elderly patients and those with refractory or relapsed disease.

## 2 Epigenetic mechanisms of drug resistance in AML

**FIGURE 1:**
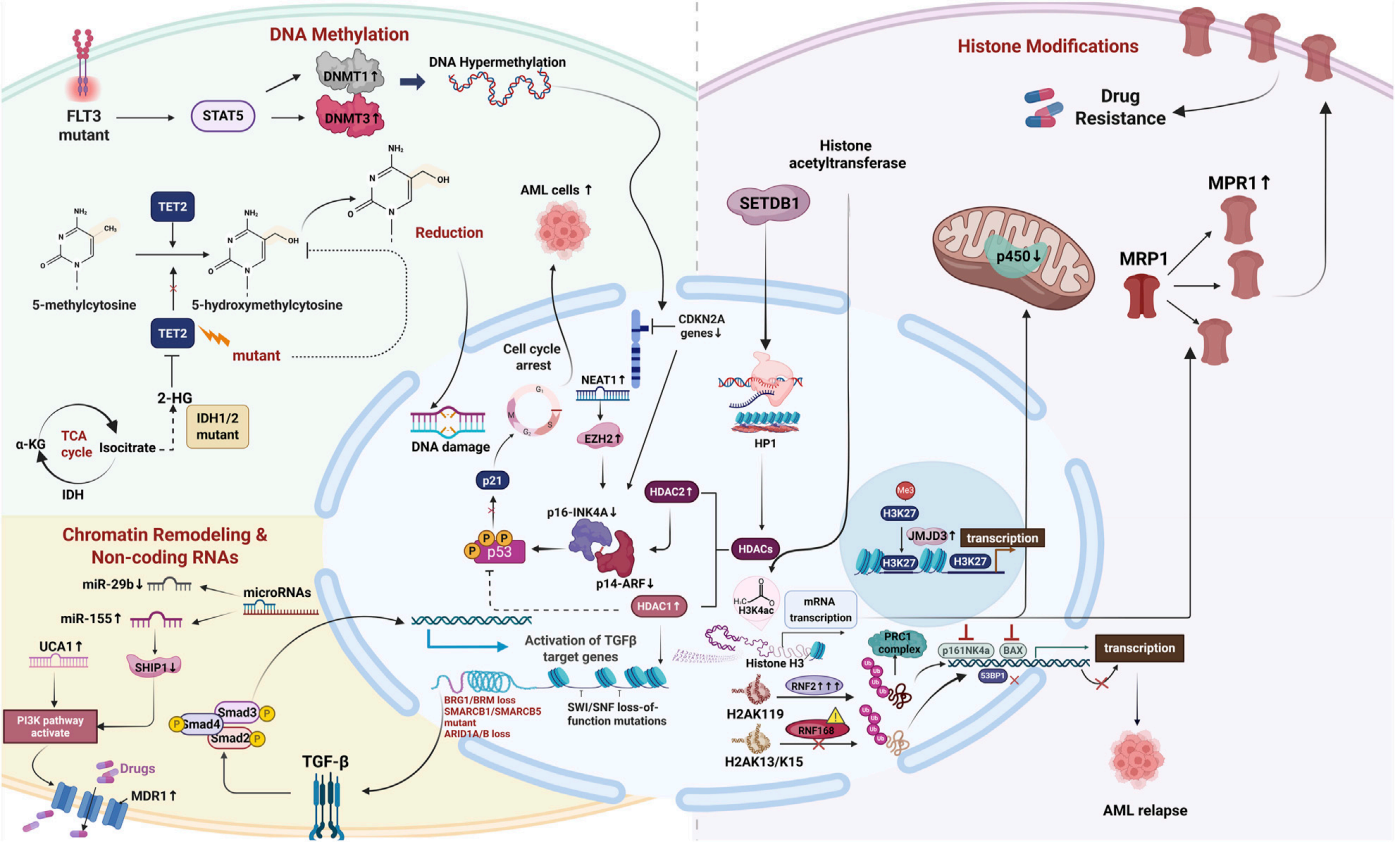
The multi-dimensional mechanism network of epigenetic regulation mediating chemotherapy resistance in AML. Illustration was created with BioRender.

Epigenetic alterations-including DNA methylation, histone modifications, chromatin remodeling, and non-coding RNA-mediated regulation, profoundly influence gene expression without altering the underlying DNA sequence. These modifications regulate essential cellular processes such as proliferation, differentiation, apoptosis, and drug response, thereby enabling leukemic cells to survive under therapeutic stress. Epigenetic dysregulation in AML plays a pivotal role in driving drug resistance by reprogramming transcriptional networks and upregulating genes associated with therapeutic resistance. The complexity and reversibility of these epigenetic mechanisms not only reinforce leukemic cell survival but also present promising targets for therapeutic intervention. The following sections will focus on the mechanistic roles of distinct epigenetic modifications in promoting drug resistance in AML (Figure 1).

### 2.1 DNA methylation

DNA methylation is a fundamental epigenetic modification that regulates gene expression by adding a methyl group to cytosine residues, primarily at CpG islands within gene promoter regions (Ordonez et al., 2019). In AML, aberrant DNA methylation manifests as both promoter hypermethylation and global hypomethylation. Promoter hypermethylation of tumor suppressor genes, such as *TP53*, *CDKN2A*, and *MLH1*, leads to transcriptional silencing, contributing to leukemogenesis and resistance to apoptosis. Conversely, DNA hypomethylation, often associated with DNMT3A mutations, promotes genomic instability and oncogene activation, thereby inhibiting differentiation and apoptosis while enhancing the survival and proliferation of leukemic cells (Vajen et al., 2013; Ordonez et al., 2019). In addition, aberrant DNA demethylation has been shown to promote chemotherapy resistance by reactivating oncogenes and suppressing tumor suppressor pathways, enabling leukemic cells to evade treatment-induced apoptosis (Cimmino et al., 2017). Collectively, these methylation-related alterations are closely linked to drug resistance in AML. In the following section, we summarize key genes and regulatory enzymes involved in DNA methylation that are specifically associated with therapy resistance.

#### 2.1.1 DNA methyltransferases (DNMTs)

DNA methylation is one of the earliest discovered and most extensively studied epigenetic modifications. It is primarily catalyzed by DNMTs, which mediate the addition of a methyl group to the fifth carbon of cytosine residues, particularly within CpG dinucleotide regions. This modification is typically associated with transcriptional repression and plays a critical role in maintaining gene expression stability, regulating cell fate decisions, and preserving chromosomal integrity (Bolouri et al., 2018).

The DNMT family comprises three main members: DNMT1, DNMT3A, and DNMT3B, which have distinct functions in maintaining or establishing DNA methylation patterns. DNMT1 is primarily responsible for maintaining methylation during DNA replication and is frequently upregulated in AML, where it contributes to persistent silencing of genes such as *TP53* and *CDKN2A*, thereby impairing apoptosis and attenuating DNA damage responses (Ling et al., 2023). DNMT3A and DNMT3B are responsible for *de novo* DNA methylation. DNMT3A R882 mutants show significant resistance to anthracyclines (Shabashvili et al., 2022), while TET2 loss reduces HMA response rates by 40% (Aldoss et al., 2020). Emerging assays must guide therapeutic decisions: Remethylation dynamics (genome-wide methylation reshaping speed post-DNMTi correlates with OS) and dsRNA/interferon signatures (patients with high IFN signals achieve 54% ORR with PD-1 inhibitors) are critical new tools (Chiappinelli et al., 2015; Saxena et al., 2021). These demand trial redesign: IDH1/2 studies should monitor 2-HG clearance thresholds (<35 nM), and DNMT3A-mutant cohorts require adaptive designs (e.g., I-SPY2 model) assigning hypomethylation responders to high-dose SGI-110. Clinically, DNMT3A mutations are associated with poor prognosis and resistance to anthracycline-based chemotherapy, such as daunorubicin (Shabashvili et al., 2022). Although DNMT3B mutations are less frequent, DNMT3B plays a vital role in sustaining the epigenetic landscape of LSCs and regulating gene networks involved in relapse and treatment resistance (Schulze et al., 2016).

In addition, DNMTs promote AML chemoresistance by altering both gene expression and signaling activity. The TP53/BCL-2 axis represents a key epigenetically regulated mechanism contributing to chemoresistance in AML. Hypomethylation of the BCL-2 promoter leads to its overexpression, which impairs TP53-mediated apoptotic signaling and supports leukemic cell survival. Treatment with DNMT inhibitors such as 5-azacytidine and decitabine restores BCL-2 methylation, reactivating apoptotic pathways and improving chemosensitivity (Wong et al., 2019; Carter et al., 2023). DNMT3A also represses CDKN2A, reducing p16-INK4A and p14ARF expression, thereby impairing p53 signaling and enabling unchecked proliferation (Ng et al., 2011). Similarly, hypomethylation of the MDR1 promoter region promotes the expression of P-glycoprotein, which actively exports chemotherapeutic agents and reduces intracellular drug accumulation (Lourenco et al., 2008). In the JAK/STAT5 axis, FLT3-ITD mutations activate STAT5, which upregulates DNMT1 and DNMT3A, driving global promoter hypermethylation and promoting LSC persistence. DNMT-mediated repression of transcription factors such as HOXA9 and GATA2 further supports LSC quiescence and immune evasion (Chen et al., 2025). Furthermore, DNMT-dependent silencing of Wnt pathway antagonists, including DKK1, SFRP1, and WIF1, facilitates sustained Wnt/β-catenin signaling, which has been linked to chemoresistance and disease persistence (Simon et al., 2005; Wong et al., 2019).

#### 2.1.2 TET proteins

The TET family of enzymes, consisting of TET1, TET2, and TET3, facilitates active DNA demethylation by catalyzing the oxidation of 5-methylcytosine (5 mC) to 5-hydroxymethylcytosine (5hmC), a critical intermediate in DNA demethylation. This process plays an essential role in regulating gene expression programs involved in hematopoietic differentiation, apoptosis, and immune function (Wong et al., 2019). Among the TET enzymes, TET2 is the most frequently mutated in AML, with loss-of-function variants observed in 15%–20% of patients. These mutations result in diminished 5hmC levels and widespread DNA hypermethylation, leading to transcriptional repression of key regulators of differentiation, DNA repair, apoptosis, and immune surveillance (Li et al., 2023). Functionally, TET2 deficiency enhances the self-renewal capacity of LSCs, impairs terminal differentiation, and confers reduced sensitivity to both cytotoxic agents and hypomethylating agent (HMAs). Additionally, TET2 mutations are associated with primary and secondary resistance to HMAs such as azacitidine and decitabine, which rely on functional TET activity to induce DNA hypomethylation (Aldoss et al., 2020; Pelosi et al., 2022).

Apart from the influence of genetic mutations, TET proteins are also influenced by regulatory metabolites (Jiang et al., 2019). A critical cofactor required for TET enzymatic activity is IDH, especially for IDH2. Although IDH is not traditionally classified as an epigenetic regulator, its mutations-most notably IDH1-R132H and IDH2-R140Q, have profound effects on epigenetic modifications, particularly in AML. Typically, isocitrate, a key intermediate in the tricarboxylic acid (TCA) cycle, is normally converted to α-ketoglutarate (α-KG) by IDH. α-KG serves as a crucial co-factor for TET2, supporting its role in DNA demethylation (Kunadt et al., 2022). Mutations in IDH1/2 lead to the neomorphic production of 2-hydroxyglutarate (2-HG), which inhibits TET2 activity, disrupts DNA demethylation, and contributes to epigenetic dysregulation in AML (Keum and Choi, 2015; Fujii et al., 2016; Gabellier et al., 2025). This inhibition further reduces 5hmC levels and contributes to promoter hypermethylation at genes involved in myeloid differentiation (GATA2, HOXA9), apoptosis (BIM, BAX), and tumor suppression (TP53, CDKN2A), thereby promoting leukemogenesis and therapeutic resistance. Notably, co-mutations in TET2 and IDH1/2 in AML patients lead to a synergistic disruption of DNA demethylation. This combined epigenetic inactivation results in more pronounced promoter hypermethylation and transcriptional silencing, further reinforcing leukemic transformation and diminishing response to both HMAs and conventional chemotherapy (Rahmani et al., 2018; Ling et al., 2023; Lo et al., 2023).

### 2.2 Histone modifications

Histone modifications, including acetylation, methylation, phosphorylation, and ubiquitination, serve as critical epigenetic regulators and are intricately involved in the development of drug resistance and the progression of AML by modulating chromatin architecture and gene expression. Furthermore, histone modifications are closely linked to the function of leukemia stem cells, which are often the primary source of chemotherapy resistance. LSCs maintain self-renewal and uncontrolled proliferation through abnormal histone modifications, contributing to the persistence of leukemia and its resistance to treatment. Given their pivotal role, understanding histone modification mechanisms is crucial for identifying novel therapeutic targets and developing more effective treatment strategies. The following sections will develop into specific histone modifications and their contributions to AML drug resistance (Asada and Kitamura, 2019; Dhall et al., 2019; Illiano et al., 2020; Xu et al., 2022).

#### 2.2.1 Histone acetylation

Both histone acetylation and deacetylation are pivotal in regulating gene expression, significantly influencing chemotherapy resistance in AML. The balance between acetylation and deacetylation is crucial for maintaining the appropriate chromatin structure for effective gene expression regulation.

Histone acetylation at lysine residues such as H3K9, H3K27, and H4K16 neutralizes their positive charge, loosening chromatin and facilitating the transcription of genes implicated in stemness (e.g., HOXA cluster), ATP-binding cassette (ABC) drug transporters, DNA repair, and anti-apoptotic pathways, collectively underpinning AML cell resistance to chemotherapeutic and targeted agents (Wen et al., 2018). This dynamic acetylation–deacetylation equilibrium enhances chromatin plasticity, enabling rapid activation of adaptive survival circuits such as MAPK, mTOR, and p53 signaling under treatment stress and within the bone marrow niche (Fujii et al., 2016). Moreover, AML stem cell subpopulations preserve specific acetylation marks like H3K14ac and H4K16ac to sustain stem cell gene expression and attenuate drug influx (e.g., via ENT1 transporter modulation), thereby diminishing the efficacy of agents like cytarabine (Xu et al., 2022). Ultimately, these epigenetic adaptations promote the selection and expansion of resistant leukemic clones under therapeutic pressure, suggesting that interventions modulating histone acetylation dynamics could represent a viable avenue to overcome AML resistance.

Additionally, histone acetylation represents a commonly occurring post-translational modification mediated by HATs, such as p300 and CBP, which catalyze the transfer of an acetyl group from acetyl-CoA to lysine residues on the histone tail. This modification neutralizes the positive charge of lysines, leading to a more relaxed chromatin structure that facilitates the binding of transcription factors to DNA and thus enhances gene transcription (Shvedunova and Akhtar, 2022). This promotes expression of oncogenes including MYC, HOXA9, and MEIS1, which sustain AML cell proliferation and block differentiation (Au et al., 2021; Zhang et al., 2022). Besides, HATs support AML cell proliferation and potentially contribute to chemotherapy resistance by influencing transcription factors that activate survival pathways (Takao et al., 2021; Zheng et al., 2024). Mutations in AML are associated with aberrant histone acetylation, whose mutations can alter lysine demethylase (KDM) activity and enhance histone deacetylation, leading to the accumulation of 2-HG. This oncometabolite inhibits HDAC activity, suppressing apoptosis-related genes and further enhancing drug resistance (Tretbar et al., 2024; Zheng et al., 2024). These interrelated mechanisms further highlight the critical role of histone modification in the pathogenesis of AML and its contribution to therapeutic resistance. What’s more, the BET family of proteins (BRD2/3/4) functions as epigenetic “readers” that bind acetylated lysine residues on histone tails and recruit the Mediator and P-TEFb complexes to facilitate both transcriptional initiation and elongation by RNA polymerase II. AML is frequently driven by oncogenic super-enhancers—for example, at the MYC locus—which exhibit high dependency on BRD4. Administration of the BET inhibitor selectively displaces BRD4 from these super-enhancer domains, resulting in downregulation of key oncogene expression, induction of leukemic differentiation, and apoptosis in AML cells. In models harboring MLL rearrangements or NPM1 mutations, sensitivity to BET inhibition is notably enhanced. Moreover, BRD4 has been implicated in modulating reactive oxygen species levels and autophagy pathways, thereby influencing leukemic cell survival. AML cells also demonstrate a global reduction in H3 acetylation, partially attributable to oncogenic fusion proteins (e.g., PML-RARα, AML-ETO), aberrantly recruiting HDACs. Therefore, combination strategies that pair BET inhibitors with DNMT or HDAC inhibitors may restore acetylation-dependent transcriptional regulation, improve clinical responses, and overcome resistance to BET therapy.

In summary, a therapeutic rationale is suggested by targeting p300/CBP to restrict the proliferation of AML, providing a multi-pronged epigenetic strategy for improving AML treatment outcomes.

#### 2.2.2 Histone deacetylation

Histone deacetylation leads to the removal of acetyl groups from lysine residues on histone tails, restoring their positive charge, reinforcing histone–DNA affinity, and promoting a more compact, transcriptionally repressed chromatin state. In AML, this compaction silences tumor-suppressor and differentiation-associated genes, thereby preserving leukemic gene expression programs and blocking myeloid cell differentiation (Wouters and Delwel, 2016). Extensive genome-wide profiling of AML blasts reveals widespread reduction of histone H3 acetylation at promoter regions, correlating with transcriptional repression of genes such as PRDX2, whose silencing is associated with poorer prognosis and unchecked leukemic proliferation. Moreover, this chromatin-condensed state supports AML cell resistance by dampening apoptotic pathways, enhancing DNA repair mechanisms, and reinforcing drug-efflux and survival circuits—thus enabling neoplastic clones to evade cytotoxic and targeted therapies (Akbarzadeh et al., 2024). These epigenetic changes also contribute to cellular quiescence within stem-like AML subpopulations, facilitating their persistence under treatment pressure (Akbarzadeh et al., 2024). The resulting selective advantage allows refractory clones to emerge, underscoring the importance of targeting the balance between acetylated and deacetylated chromatin states to overcome therapeutic resistance in AML.

Histone deacetylation, mediated primarily by HDACs and sirtuins, represses transcription by compacting chromatin, thereby silencing tumor-suppressor genes and differentiation programs in AML cells. Aberrant recruitment of histone deacetylase complexes by oncoproteins such as AML1–ETO and PML–RARA enforces chromatin compaction at promoters of tumor-suppressor and differentiation-related genes, thereby preserving oncogenic transcriptional programs, preventing normal myeloid maturation, and fostering chemotherapeutic resistance in AML. Class I HDACs—particularly HDAC1, HDAC2, and HDAC3—are frequently overexpressed in AML and form corepressor complexes such as NuRD and Sin3A, which deacetylate histones at promoters of critical genes like p21, p53, and BIM. This deacetylation leads to chromatin compaction and transcriptional silencing of these tumor suppressors, thereby promoting uncontrolled proliferation and resistance to apoptosis (Amin et al., 2023; Gu et al., 2023). Histone deacetylation, primarily catalyzed by HDACs, is a key epigenetic modification that regulates chromatin condensation and transcriptional repression in AML. HDAC2 contributes to drug resistance in AML by repressing tumor suppressor genes such as p16INK4a and p21, thereby promoting unchecked cell proliferation and survival (Kim et al., 2013; Zheng et al., 2024). Similarly, HDAC3 also represses genes involved in DNA damage response and cell cycle checkpoints, enabling AML cells to evade genotoxic stress induced by chemotherapy (Dai et al., 2023). HDAC6 also deacetylates non-histone substrates, including α-tubulin and HSP90, which stabilizes oncogenic client proteins such as FLT3-ITD and c-MYC, enhancing leukemic cell survival under chemotherapeutic pressure (Li et al., 2018; Carbajo-Garcia et al., 2022).

In summary, dysregulated histone deacetylation establishes an epigenetic environment conducive to AML progression and therapeutic resistance. Targeting HDACs offers a promising approach to reprogram chromatin states, restore tumor suppressor function, and improve AML treatment outcomes.

#### 2.2.3 Histone methylation

Histone methylation is an important epigenetic modification that regulates gene expression by adding methyl groups to specific lysine residues on histones. These modifications influence gene activity based on the specific histone residue and the type of methylation. For example, trimethylation of H3K4 (H3K4me3) is commonly associated with transcriptionally active regions, whereas trimethylation of H3K27 (H3K27me3) is associated with transcriptional repression. In AML, alterations in histone methylation can impact the expression of critical tumor suppressor genes and contribute to drug resistance (Lei et al., 2018).

A histone methyltransferase, EZH2, catalyzes the trimethylation of H3K27, leading to changes in gene expression. In AML, overexpression of EZH2 results in the downregulation of tumor suppressor genes, such as *p16INK4a* and *p53*, which promotes leukemia cell proliferation and increases resistance to chemotherapy (Huang et al., 2021). Another important modification is H3K9 trimethylation (H3K9me3), which is catalyzed by SUV39H1 and SETDB1. In AML, elevated levels of H3K9me3 contribute to the repression of tumor suppressor genes, enhancing drug resistance and enabling leukemia cells to evade chemotherapy-induced apoptosis. Overexpression of SUV39H1 and SETDB1 in AML further strengthens these effects, facilitating the progression of the disease and resistance to treatment. In contrast, H3K4me3, a hallmark of transcriptionally active regions, is commonly associated with gene activation. Mutations in the ALL gene that lead to dysregulation of H3K4me3 levels have been implicated in AML resistance by activating oncogenes and enhancing chemotherapy drug resistance (Massett et al., 2021; Tsai et al., 2022).

#### 2.2.4 Histone demethylation

Histone demethylases play a crucial role in regulating chromatin structure and gene expression by removing methyl groups from histones. These modifications can alter chromatin accessibility and gene activity, influencing processes such as drug resistance in cancer. Specific demethylation events can reverse the repression of gene expression, thereby impacting the mechanisms that contribute to chemoresistance in AML.

Jumanji Domain-Containing Protein 3 (JMJD3), a key histone demethylase, plays an essential role in regulating gene expression by removing the H3K27me3 mark. In AML, overexpression of JMJD3 has been shown to correlate with increased chemotherapy resistance. This occurs through the removal of H3K27me3, leading to the reactivation of genes that promote leukemic cell proliferation and survival, thereby facilitating resistance to chemotherapy-induced cytotoxicity (Rejlova et al., 2018; Zheng et al., 2023). Jumanji Domain-Containing Protein 1A (JMJD1A) is also involved in histone demethylation, specifically targeting H3K9me1 and H3K9me2. The removal of these methyl marks by JMJD1A further contributes to chemotherapy resistance by activating genes associated with cell survival (Jafek et al., 2019). Lysine-Specific Demethylase 1 (LSD1), which removes H3K4me1 and H3K9me2, is often overexpressed in AML. The overexpression of LSD1 in AML contributes to the suppression of tumor suppressor genes, thereby enhancing leukemia cell survival and increasing resistance to chemotherapy (Jafek et al., 2019; Wass et al., 2021). Additionally, IDH mutations, which have also been found to alter histone methylation patterns, lead to the accumulation of 2-HG, inhibiting the activity of histone demethylases such as JMJD3, LSD1, and JMJD1A. This inhibition results in abnormal histone methylation, which disrupts gene expression and contributes to leukemia cell proliferation and resistance to chemotherapy (Tian et al., 2022; Thomas et al., 2023; Zhu et al., 2024).

#### 2.2.5 Histone phosphorylation

Histone phosphorylation serves as a critical regulatory mechanism implicated in DNA repair, cell cycle progression, and anti-apoptotic pathways, particularly in the context of drug resistance in AML. The histone variant H2AX is central to the DNA damage response, especially during double-strand breaks. Over-phosphorylation of H2AX in AML cells enhances DNA repair activity, correlating with increased resistance to chemotherapy drugs (Cao et al., 2016). Additionally, the Mitogen-Activated Protein Kinase (MAPK) pathway mediates phosphorylation of histone H3 at serine 10 (H3S10) in AML cells. This activation promotes cell proliferation and inhibits chemotherapy-induced apoptosis, further contributing to drug resistance (Liu et al., 2023; Qiu et al., 2024). Additionally, Aurora Kinases (AURKA and AURKB) phosphorylate H3 at serines 10 and 28 (H3S10 and H3S28), promoting mitotic progression and cell cycle advancement. Together, these phosphorylation events form a complex epigenetic network that drives AML cell survival and therapeutic resistance (Park et al., 2018).

#### 2.2.6 Histone ubiquitination

Histone ubiquitination is a critical epigenetic modification that regulates chromatin remodeling and gene expression, playing a significant role in drug resistance in AML. RING Finger Protein 2 (RNF2), an E3 ubiquitin ligase, mediates monoubiquitination of histone H2A at lysine 119 (H2AK119ub), resulting in chromatin compaction and transcriptional silencing of tumor suppressor genes. Overexpression of RNF2 in AML cells is associated with enhanced drug resistance by repressing genes critical for chemotherapy response (Yamamoto et al., 2014; Shima et al., 2018). Similarly, ubiquitin-like with PHD and RING finger domains 1 (UHRF1), a multifunctional protein, facilitates histone H3 ubiquitination and coordinates DNA methylation. Elevated UHRF1 expression in AML cells drives aberrant gene expression patterns, contributing to chemotherapy resistance (Simonetti et al., 2019). These ubiquitination events collectively highlight a complex epigenetic regulatory network in AML, offering potential therapeutic targets to overcome drug resistance.

### 2.3 Chromatin remodeling

Chromatin remodeling, a fundamental epigenetic process, dynamically alters chromatin structure to regulate DNA accessibility and gene expression, playing a critical role in chemotherapeutic resistance in AML. Dysregulation of chromatin remodeling complexes, such as SWI/SNF, ISWI, and CHD, influences critical processes like DNA repair, cell cycle progression, apoptosis, and stem cell-like traits, all of which contribute to chemoresistance.

In the SWI/SNF complex, BRG1 (SMARCA4) and BRM (SMARCA2) act as ATPases that reorganize chromatin and support DNA repair; their loss disrupts normal gene expression, increasing AML cell resistance to chemotherapy (Chambers et al., 2023; Yang et al., 2025). SMARCB1 maintains chromatin in an open state and activates tumor suppressor genes. Similarly, mutations in SMARCB1 may inhibit apoptosis and increase the risk of chemoresistance (Chatterjee et al., 2018). ARID1A/ARID1B are involved in chromatin positioning and DNA damage response, and their deletion may lead to impaired DNA damage response, promoting chemoresistance via TGF-β1/SMAD3 signaling (Stratmann et al., 2021; Wang et al., 2024). In the ISWI complex, SMARCA5 is involved in regulating DNA replication and transcription, and regulating the differentiation of leukemic cells. Mutations in SMARCA5 can cause chromatin condensation, reducing the nuclear permeability of chemotherapeutic agents (Dluhosova et al., 2014; Jevtic et al., 2022).

### 2.4 Non-coding RNA regulation

Non-coding RNAs (ncRNAs) are critical regulators of gene expression and chromatin structure in AML, particularly in the development of drug resistance. Dysregulated ncRNAs, including microRNAs (miRNAs), long non-coding RNAs (lncRNAs), and circular RNAs (circRNAs), disrupt cellular homeostasis and reduce treatment efficacy. For instance, miR-155 promotes resistance to cytarabine and anthracyclines by targeting tumor suppressors TP53INP1 and SHIP1 to inhibit apoptosis via PI3K/AKT signaling (Shima et al., 2018), while downregulated miR-29b fails to repress DNMT3A/B, leading to tumor suppressor hypermethylation and diminished decitabine sensitivity (Mims et al., 2013). Similarly, lncRNA UCA1 activates AKT/mTOR signaling and upregulates MDR1, conferring resistance to doxorubicin and cytarabine (Nepstad et al., 2020), and NEAT1 enhances DNA damage repair by recruiting EZH2 for H3K27me3-mediated silencing and stabilizing ATR/CHK1 signaling (Boudny and Trbusek, 2020; Yan et al., 2021). Additionally, circPAN3 sponges miR-153-3p to upregulate BCL2, reducing cytarabine-induced apoptosis, and modulates RNA polymerase II activity to reshape the leukemic transcriptome (Shang et al., 2019; Li et al., 2020). This intricate ncRNA network drives AML chemoresistance, highlighting the therapeutic potential of targeting miR-155, UCA1, or circPAN3 with antisense oligonucleotides or small-molecule inhibitors to restore treatment sensitivity and improve clinical outcomes.

## 3 Therapeutic strategies of epigenetic drugs in AML

AML has witnessed significant therapeutic advancements in recent years due to a deeper understanding of epigenetic regulation. Epigenetic therapies targeting DNA methylation, histone modification, and metabolic dysregulation provide precision treatment options for molecularly defined AML subtypes (Stein et al., 2021; Ravandi et al., 2024). Although epigenetic monotherapies such as low-dose DNA methyltransferase inhibitors (azacitidine/decitabine) and bromodomain, have demonstrated the ability to eradicate chemotherapy-induced senescent AML subsets, deplete leukemic stem-like cells, and enhance initial responses in refractory AML, their efficacy as stand-alone regimens is significantly limited. This is primarily due to intrinsic and adaptive transcriptional plasticity, which enables rapid compensatory reprogramming (e.g., p300-mediated enhancer rewiring following BET inhibition), heterogeneous resistance mechanisms, including DNMT1 downregulation or deletion attenuating sensitivity to DNMTs inhibitors, drug influx transporter loss, and secondary mutations in epigenetic regulators, and off-target cytotoxicity and hematologic toxicity, which constrain dosage and continuous administration (Shah et al., 2025). Primary resistance (e.g., low response rates in TET2/IDH wild-type patients treated with DNMTi) and acquired resistance (e.g., secondary mutations after IDH inhibitor therapy) require deeper molecular characterization (Lee et al., 2019; McMurry et al., 2021). A critical therapeutic challenge lies in the delayed clinical response (median time to response: 8–12 weeks), which contrasts starkly with the rapid disease progression observed in AML. This kinetic discrepancy has spurred investigations into rational combination strategies, particularly with BCL-2 inhibitors (e.g., venetoclax) or immune checkpoint modulators, to accelerate therapeutic efficacy and mitigate early treatment failure (DiNardo et al., 2019; DiNardo et al., 2020). Collectively, these insights underscore a broader imperative: the design of epigenetic combination regimens must be anchored in specific underlying chromatin- and transcription-based resistance mechanisms present in AML. For instance, persistent H3K27me3 marks—which reflect transcriptional repression of tumor suppressor genes mediated by EZH2/PRC2 activity—provide a direct mechanistic rationale for combining EZH2 inhibitors with DNA-demethylating agents. Studies have demonstrated that co-inhibition of EZH2 and DNMTs leads to widespread epigenomic reprogramming, reactivation of apoptotic and cell cycle regulatory genes, and enhanced cytotoxicity in cancer models (Atienza Parraga et al., 2025).

Likewise, the transcriptional rebound observed following BET inhibition in AML has been mechanistically linked to a compensatory feedback loop mediated by the histone acetyltransferase p300. Specifically, BET inhibition (e.g., BRD4 displacement) triggers p300-dependent restoration of transcription at AML-maintaining genes—attenuating initial gene downregulation. Sequential application—first deploying BET inhibitors to shut down oncogenic transcription, followed by p300 inhibition—has been shown preclinically to disrupt this compensatory mechanism, sustain repression of key drivers like MYC, and enhance synergistic cytotoxicity ASH Publications (Wang and Vakoc, 2025). By explicitly linking mechanistic biomarkers (e.g., H3K27me3 persistence, p300-mediated transcriptional rebound) to targeted interventions (EZH2 + DNMT inhibition; sequential BET→p300 inhibition), therapeutic designs can achieve tighter conceptual flow—and thus maximize synergy, overcome adaptive resistance, and potentially reduce toxicity. Future research should focus on overcoming resistance mechanisms and optimizing combination strategies to enhance clinical outcomes.

### 3.1 Monotherapy for resistance in AML

Epigenetic regulatory drugs have demonstrated significant clinical value in the monotherapy of AML drug resistance. Azacitidine and decitabine are the most common HMAs, which hold critical importance as standard first-line monotherapy for newly diagnosed AML patients who are elderly or deemed unfit for intensive “7 + 3” induction chemotherapy. Multiple prospective trials demonstrate that these HMAs significantly improve complete remission rates (CRR), transfusion independence, and median OS compared to supportive care or low-dose cytarabine. As monotherapy, they typically extend survival to approximately 7–11 months in this specific patient population (Lee et al., 2021). However, their efficacy as single agents is limited by rapid enzymatic degradation (decitabine t_1__/2_ ≈ 35–47 min; azacitidine t_1__/2_ ≈ 20–41 min) (Jabbour et al., 2013; Lee et al., 2021; Wang et al., 2021). On the other hand, azacitidine and decitabine are more available in parenteral form, requiring patients to come to a treatment center daily for 5 or 7 consecutive days of every 28-day treatment cycle and imposing a substantial burden on the largely older adult population affected by AML.

To prolong the duration and reduce the burden on the patient, next-generation HMAs such as guadecitabine (SGI-110), oral azacitidine (CC-486), and oral decitabine-cedazuridine (DEC-C) are under clinical evaluation (Garcia-Manero et al., 2022; Efficace et al., 2024; Garcia-Manero et al., 2024a). SGI-110, a second-generation DNMTi, is a dinucleotide conjugate of decitabine and deoxyguanosine that resists degradation by cytidine deaminase, conferring prolonged *in vivo* exposure and enhanced clinical activity (Haggarty et al., 1988). A phase III trial involving 606 elderly AML patients demonstrated reduced risk of health-related quality of life (HRQoL) deterioration at 2 months with SGI-110 *versus* intensive chemotherapy (76% vs. 88%), suggesting survival benefits (Efficace et al., 2024). Its pharmacokinetic stability and favorable safety profile position SGI-110 as a promising candidate for combination therapies in myeloid malignancies (Haggarty et al., 1988). Oral azacitidine (CC-486) represents a hypomethylating agent that can be administered on a prolonged dosing schedule to sustain therapeutic activity, which has received FDA and EMA approval as maintenance therapy for adult AML patients who are ineligible for hematopoietic cell transplantation (HCT). The pharmacokinetic and pharmacodynamic profiles of CC-486 are distinct from injectable azacitidine formulations (Gaudy et al., 2023). Moreover, previous studies have demonstrated the sustained efficacy of CC-486 in patients who have developed resistance to demethylating drug injection preparations. A phase III trial (Wei et al., 2020) in AML patients undergoing maintenance therapy post-initial induction remission demonstrated superior OS (24.7 vs. 14.8 months; P < 0.001) and relapse-free survival (RFS: 10.2 vs. 4.8 months; P < 0.001) for CC-486 *versus* placebo, with favorable tolerability. Oral decitabine/cedazuridine (DEC-C) achieved comparable pharmacokinetics and median OS to intravenous DAC in a randomized crossover study of 89 chemotherapy-ineligible AML patients (Geissler et al., 2024). DEC-C enables home-based therapy, and thus may reduce treatment burden and improve adherence, which would be of particular value in older patient. The fixed-dose combination of oral Decitabine and Cedazuridine (INQOVI^®^) has been approved by FDA and the European Union for treating AML patients ineligible for standard induction chemotherapy due to comorbidities. SGI-110, IDH305, and EPZ-5676 have also demonstrated potent anti-tumor efficacy in preclinical and early trials but remain in clinical development due to mandatory multi-phase validation. This requires rigorous assessment of long-term safety, efficacy consistency in larger cohorts, optimal dosing, and compliance with regulatory standards for approval. Cancer drugs necessitate thorough evaluation to mitigate unforeseen risks in complex biological systems.

While innovations in HMA delivery address critical challenges of tolerability and convenience, concurrent progress is being made in targeting specific molecular vulnerabilities within AML. Ivosidenib and enasidenib, novel mutant-selective IDH1 inhibitors, suppress neomorphic enzyme activity to reduce oncogenic 2-HG accumulation, thereby restoring physiological DNA methylation patterns (Lee et al., 2019). These agents are FDA-approved as first-line therapy for adults with IDH-mutated relapsed/refractory (R/R) AML. Clinical outcomes reveal Ivosidenib monotherapy achieves CR/CRi rates of 63% and 72% respectively, in IDH1-mutated R/R AML, with an estimated 1-year OS of 76%–78% (Stein et al., 2021). Phase I data for enasidenib combined with azacitidine demonstrate a CR rate of 57% (Stein et al., 2021), a CR + CRi rate of 70%, and favorable tolerability and safety profiles. IDH305, a brain-penetrant allosteric IDH1 inhibitor, showed preclinical efficacy but was discontinued due to a narrow therapeutic window (DiNardo et al., 2023).

Despite extensive research into epigenetic mechanisms driving AML resistance—including histone modifications, chromatin remodeling, and non-coding RNA dysregulation—single-agent therapies targeting these pathways face significant challenges. The high complexity and redundancy of epigenetic networks allow compensatory pathways to activate when one node is inhibited (e.g., EZH2 suppression triggering acetyltransferase upregulation). Tumor heterogeneity and epigenetic plasticity further enable the selection of resistant subclones that evade monotherapies. Additionally, biological limitations, such as the reliance of HMAs on cell division and difficulties in targeting non-coding RNAs regularly restrict efficacy. Safety concerns also arise from the broad roles of epigenetic regulators in normal cells, exemplified by the narrow therapeutic window of HDAC or histone lysine methyltransferase (DOT1L) inhibitors. These barriers are starkly contrasted by the success of epigenetic monotherapies in other hematologic malignancies, where tazemetostat (EZH2i) achieves 69% ORR in EZH2-mutant follicular lymphoma, romidepsin (HDACi) induces 34% ORR in cutaneous T cell lymphoma, and azacitidine (DNMTi) elevates CR rates to 17%–20% in high-risk MDS (Morschhauser et al., 2020; Piekarz et al., 2011; Fenaux et al., 2009). The profound inefficacy of these same agents in resistant AML stems from disease-specific vulnerabilities: (1) Epigenetic dysregulation here is adaptive-not a primary driver—with mechanisms like ASXL1-loss-driven EZH2 upregulation (Ganan-Gomez et al., 2015); (2) Network redundancies rapidly bypass inhibition (DNMTi blockade activates EZH2-mediated repression; Chen et al., 2020); (3) Leukemia stem cells exploit niche signals (TGF-β→KDM6A; Wei et al., 2025) or hypoxia-induced SIRT1 to evade targeting; and (4) Acquired resistance mutations (e.g., DNMT3A R882; Russler-Germain et al., 2014) and noncoding RNA rewiring (HOTAIR) further undermine monotherapy.

Clinical evidence supports the use of rational combination regimens that concurrently target compensatory survival circuits and microenvironmental support in AML. For example, venetoclax combined with hypomethylating agents (DNMTi + venetoclax) has achieved CR/CRi rates exceeding 70% in patients unfit for intensive chemotherapy (DiNardo et al., 2020), Similar strategies, such as HMAs combined with TGF-β pathway inhibitors to mitigate stromal-mediated protection, have shown preclinical promise. Additionally, dual epigenetic degraders targeting both EZH2 and BRD4 demonstrate synergistic activity in preclinical models, indicative of synthetic lethality as a mechanism to enhance efficacy over monotherapy.

### 3.2 Novel combination therapy for targeting resistance in AML

In response to these challenges, increasing efforts have been devoted to exploring epigenetic-based combination therapies, which aim to target multiple epigenetic regulators or integrate epigenetic agents with other classes of anti-leukemic drugs. These combinatorial strategies are designed to overcome epigenetic redundancy, disrupt parallel resistance pathways, and synergistically enhance leukemic cell susceptibility to treatment. Beyond the theoretical rationale, mounting preclinical studies have demonstrated that combining DNMT with HDAC inhibitors or HMTs can reprogram leukemic cell epigenomes, reactivate tumor suppressor genes, and sensitize resistant blasts to apoptosis. For instance, hypomethylating agents such as azacitidine or decitabine, when paired with HDAC inhibitors, not only enhance global chromatin accessibility but also amplify pro-apoptotic signaling cascades, leading to improved therapeutic responses in otherwise refractory AML models. Furthermore, epigenetic therapies are increasingly being combined with targeted agents such as FLT3 inhibitors, BCL-2 inhibitors, or immune checkpoint modulators. These integrated approaches exploit the vulnerabilities created by epigenetic reprogramming, such as increased dependency on mitochondrial priming or altered antigen presentation, thereby enhancing drug sensitivity and immune-mediated clearance of leukemic cells. Additionally, emerging strategies involve the use of bromodomain and extraterminal domain inhibitors in combination with other chromatin-modifying drugs, which may simultaneously suppress oncogenic transcriptional programs and destabilize leukemia stem cell self-renewal.

In the following section, we will examine the current landscape of epigenetic combination therapies in AML, highlighting key preclinical findings, ongoing clinical trials, and their potential to circumvent therapeutic resistance (Tables 1, 2). Taken together, these advances underscore the growing recognition that monotherapy is rarely sufficient in the context of AML’s genetic and epigenetic heterogeneity, and that rationally designed combination regimens may hold the key to durable therapeutic responses.

**TABLE 1:** Overview of approved therapy strategies for AML.

Drug Name	Target/Mechanism	Indication	Approved Regions	NCT Number	Notable AEs
Decitabine	DNMT inhibitor	Elderly AML (≥65 years)	United States, EU, China	NCT00043381	Lower median baseline platelet count
Azacitidine	DNMT inhibitor	Elderly AML (≥65 years)	United States, EU, China	NCT00887068	Colitis, infectious (e.g., *Clostridium difficile*) - possibly related
CC-486 (QUAZAR AML-001)	DNMT inhibitor	AML maintenance therapy	United States, EU	NCT01757535	Febrile neutropenia
Chidamide	HDAC inhibitor	R/R AML	China	NCT02886559	Infection
Ivosidenib	IDH1 mutation inhibitor	IDH1-mutated R/R AML	United States, EU	NCT06717958	Febrile neutropenia
Enasidenib	IDH2 mutation inhibitor	IDH2-mutated R/R AML	United States, EU	NCT02719574	Febrile neutropenia
Decitabine + Cedazuridine	DNMT inhibitor + bioavailability enhancer	AML patients ineligible for standard induction chemotherapy	United States, EU	NCT03306264	Febrile neutropenia
Midostaurin + Daunorubicin + Cytarabine	FLT3/Aurora kinase inhibitor + chemotherapy	FLT3-mutated AML	United States, EU	NCT01477606	Febrile neutropenia

**TABLE 2:** Overview of combination therapies under clinical investigation for AML.

Drug Name	Target/Mechanism	Indication	NCT Number	Reporting Phase
Guadecitabine	DNMT inhibitor	R/R AML	NCT02920008	Phase 3 (Completed)
IDH305	IDH1 mutation inhibitor	IDH1-mutant AML	NCT04603001	Phase 1 (Active)
Pinometostat	DOT1L inhibitor	R/R AML	NCT03724084	Phase 1/2 (Terminated)
Azacitidine + Venetoclax (VIALE-A)	DNMT inhibitor + BCL2 inhibitor	Newly diagnosed elderly AML	NCT02993523	Phase 3 (Active)
Decitabine + Venetoclax	DNMT inhibitor + BCL2 inhibitor	R/R AML	NCT02203773	Phase 1 (Terminated)
Azacitidine + Lenalidomide	DNMT inhibitor + immunomodulation	Elderly AML with del (5q)	NCT01038635	Phase 1/2 (Completed)
Azacitidine + Pracinostat	DNMT inhibitor + Pan-HDAC inhibitor	Elderly AML (≥65 years)	NCT03151408	Phase 3 (Terminated)
Azacitidine + Pevonedistat (PANTHER	DNMT inhibitor + Ubiquitination pathway inhibitor	High-risk MDS/AML	NCT03268954	Phase 3 (Completed)
RO6870810 + Cytarabine + Idarubicin	BET inhibitor + chemotherapy	High-risk MDS/AML	NCT02308761	Phase 1 (Completed)
Vorinostat + Cytarabine + Daunorubicin Hydrochloride	HDAC inhibitor + chemotherapy	R/R AML	NCT01802333	Phase 3 (Completed)
Entinostat + Pembrolizumab	HDAC inhibitor + immunomodulation	AML with myelodysplasia-related changes	NCT02936752	Phase 1 (Completed)
Decitabine + Bortezomib + Pegylated liposomal doxorubicin	DNMT inhibitor + proteasome inhibitor + chemotherapy	R/R AML	NCT01736943	Phase 2 (Completed)
Panobinostat + Idarubicin + Cytarabine	HDAC inhibitor + chemotherapy	R/R AML	NCT01242774	Phase 1 (Completed)
Vorinostat + Decitabine + Cytarabine	HDAC inhibitor + DNMT inhibitor + chemotherapy	R/R AML	NCT01130506	Phase 1 (Completed)
Chidamide + Decitabine + Cytarabine + Aclarubicin + G-CSF (CD-CAG)	HDAC inhibitor + DNMT inhibitor + chemotherapy	R/R AML	NCT02886559	Phase 1/2 (Completed)
Mivebresib + Venetoclax	Pan-bromodomain and extraterminal inhibitor + BCL2 inhibitor	R/R AML	NCT02391480	Phase 1 (Completed)
Azacitidine + Enasidenib	DNMT inhibitor + IDH2 mutation inhibitor	IDH2-mutated R/R AML	NCT02677922	Phase 1/2 (Active)

#### 3.2.1 Epigenetic drug combination with chemotherapy to overcome resistance

Epigenetic drugs combined with chemotherapy have shown promising results in overcoming treatment resistance in AML by reactivating silenced tumor suppressor genes and enhancing the sensitivity of leukemic cells to cytotoxic agents. Azacitidine with low-dose cytarabine has demonstrated superior efficacy compared to cytarabine alone. In a phase III international study in elderly AML patients, azacitidine significantly improved median OS to 10.4 months, compared to 6.5 months in the conventional care group (*P* = 0.1009) (Seymour et al., 2017). Decitabine has also been evaluated in combination with the D + A regimen (daunorubicin and cytarabine). In a study involving 81 newly diagnosed non-elderly AML patients, the decitabine-containing regimen achieved a CRR of 91.4%, compared to 69.6% with D + A alone. Notably, the 2-year OS rate was significantly improved in the combination group (*P* = 0.008) (Zheng et al., 2020). HDAC inhibitors, including vorinostat and panobinostat, have also been explored in combination with chemotherapy. By modulating chromatin structure and restoring the expression of silenced genes, HDAC inhibitors can enhance chemosensitivity, particularly in relapsed, refractory, or high-risk AML cases (Maiso et al., 2009). Moreover, DOT1L inhibitor SYC-522, when in combination with mitoxantrone, a classical chemotherapeutic agent, can significantly enhance chemotherapy efficacy by inhibiting the DNA damage response, thereby increasing the sensitivity of leukemic cells to chemotherapy, while SYC-522 alone reduces the clonogenic capacity of MLL-rearranged AML cells by only approximately 50% (Liu et al., 2014). Many other similar drug combinations exist, each offering a complementary approach to mitigating AML resistance, making combination therapy a compelling strategy in overcoming treatment challenges.

#### 3.2.2 Epigenetic drug combination with targeted therapy to combat resistance

Targeted therapies have become an essential component in the treatment of AML, particularly in patients harboring actionable mutations such as BCL-2 or FLT3. However, resistance to targeted agents remains a major clinical obstacle because AML cells frequently acquire resistance through clonal evolution and compensatory epigenetic reprogramming, thereby limiting the efficacy of monotherapy approaches. Owing to their reversible nature and central role in the regulation of leukemic cell survival and plasticity, epigenetic modulators have emerged as critical agents in combination with therapeutic strategies, which effectively improve remission rates and survival outcomes. Among targeted agents, BCL-2 inhibitors-particularly venetoclax-have demonstrated the most pronounced therapeutic efficacy when combined with epigenetic modulators. The combination of venetoclax with the hypomethylating agent azacitidine has been widely adopted in clinical practice, especially for newly diagnosed AML patients who are ineligible for intensive chemotherapy (DiNardo et al., 2019). Venetoclax exerts its anti-leukemic effect by promoting apoptosis through the mitochondrial pathway. However, secondary resistance can arise via upregulation of anti-apoptotic proteins such as MCL-1 or BCL-XL in AML cells. The VIALE-A phase III clinical trial demonstrated that the combination of azacitidine and venetoclax significantly improved clinical outcomes in elderly or unfit AML patients, achieving a median OS of 14.7 months compared to 9.6 months with azacitidine alone. Furthermore, the CR rate was markedly enhanced from 28.3% to 66.4% with the combination therapy (DiNardo et al., 2020). Furthermore, the combination of BET inhibitors and venetoclax exerts dual inhibition on the MYC/BCL-2 axis, effectively reversing apoptosis resistance. Notably, a study evaluating the BET inhibitor INCB054329 in combination with venetoclax demonstrated a marked reduction in cell viability across AML cell lines and primary patient-derived samples. The combinatorial regimen significantly suppressed leukemic burden in murine xenograft models, with no apparent treatment-related toxicity observed (Ramsey et al., 2021).

Besides, in IDH1/2-mutant AML, the differentiation-inducing effects of IDH inhibitors (e.g., ivosidenib, enasidenib) can be potentiated by co-treatment with hypomethylating agents. Azacitidine has been shown to relieve epigenetic silencing and enhance transcriptional reactivation of differentiation programs. A clinical trial combining azacitidine with ivosidenib in newly diagnosed IDH1-mutant AML reported that the estimated probability that a patient would remain event-free at 12 months was 37% in the ivosidenib-and-azacitidine group and 12% in the placebo-and-azacitidine group. The median OS was 24.0 months with ivosidenib and azacitidine and 7.9 months with placebo and azacitidine (hazard ratio for death, 0.44; 95% CI, 0.27 to 0.73; *P* = 0.001) (Montesinos et al., 2022). Likewise, azacitidine plus enasidenib yielded a CR rate of 53% in R/R IDH2-mutant AML, compared to 12% with azacitidine alone (DiNardo et al., 2021). Another phase II study in R/R IDH2-mutated AML patients has demonstrated a CR rate of 53% when utilizing azacitidine and enasidenib, compared to 12% with azacitidine alone (DiNardo et al., 2021), which significantly improved therapeutic effect. Moreover, although targeted inhibition with agents such as midostaurin and gilteritinib can treat FLT3-mutated acute AML, drug resistance frequently emerges, mainly driven by activation of alternative signaling pathways and epigenetic adaptations, including chromatin remodeling. The combination therapy of Midostaurin with Daunorubicin and Cytarabine has been approved by the U.S. FDA and the European Union for treating FLT3-mutated AML, establishing it as a standard regimen for this specific patient population. Emerging evidence suggests that DNMT inhibitors, such as azacitidine, can attenuate FLT3-associated downstream signaling cascades, particularly the PI3K/AKT pathway, thereby enhancing the sensitivity of leukemic cells to FLT3 inhibition (Tecik and Adan, 2022). A phase I/II clinical study evaluating azacitidine with gilteritinib in R/R FLT3-mutant AML showed manageable toxicity and promising early efficacy (Wang et al., 2022). Other less common epigenetic-based combinations include BET inhibitors combined with FLT3 inhibitors, as well as DOT1L or menin inhibitors used alongside hypomethylating agents in the treatment of MLL-rearranged AML. Collectively, these combination strategies demonstrate the potential of epigenetic agents to enhance the efficacy and durability of targeted therapies, providing a rational approach to overcoming acquired resistance in AML.

#### 3.2.3 Epigenetic drug combination with immunotherapy to address immune resistance

Despite the significant advances of immunotherapy in various hematologic malignancies in recent years, its efficacy in AML remains limited. This is primarily attributed to the low mutational burden of AML, impaired antigen presentation, and the presence of immunosuppressive mechanisms within the bone marrow microenvironment. Epigenetic therapies offer a promising approach to overcome these barriers by reshaping the tumor immune microenvironment, restoring antigen expression, and enhancing T cell activity, thereby opening new avenues for the application of immunotherapy in AML. The joint application of epigenetic regulation and immunotherapy can effectively alter the immune microenvironment in AML, improving therapeutic outcomes and providing a new strategy to overcome drug resistance in AML, which shows significant potential in overcoming resistance in AML treatment. Combining epigenetic modulators with immunotherapy not only boosts immune responsiveness but also provides a novel approach to bypass immune evasion and overcome drug resistance. Encouraging results from preclinical studies and ongoing clinical trials highlight the potential of this strategy to improve outcomes in drug-resistant AML.

DNMT inhibitors have become an essential therapeutic option for elderly patients with AML or those unfit for intensive chemotherapy. However, their clinical efficacy remains limited by high relapse rates and short durations of remission (Kantarjian et al., 2024). In recent years, immune checkpoint inhibitors (ICIs), such as anti-PD-1/PD-L1 antibodies, have demonstrated remarkable efficacy in various solid tumors and are now being actively explored in the treatment of hematologic malignancies (Bilgihan et al., 2024). Preclinical studies suggest that DNMT inhibitors can enhance tumor immunogenicity by upregulating cancer-testis antigens and major histocompatibility complex (MHC) molecules, as well as activating interferon signaling pathways (Chen et al., 2020). These effects help reshape the immunosuppressive tumor microenvironment in AML and potentiate the anti-leukemic activity of ICIs. Based on these findings, the combination of DNMT inhibitors and ICIs has emerged as a promising strategy to overcome therapeutic resistance in AML and improve clinical outcomes, attracting increasing research interest in recent years.

The combination of azacitidine and the PD-1 inhibitor, nivolumab, yielded an objective response in 20 patients (87%), including 17% with a complete response and 70% with a partial response according to a clinical study (Ansell et al., 2015). Immunohistochemical analyses further revealed a marked increase in CD8^+^ T cell infiltration and upregulation of PD-L1 expression in responders before and after treatment, suggesting that the therapeutic effect of this combination strategy is mediated through reactivation of the tumor immune microenvironment. In one phase Ib/II study, 19 patients with R/R AML received combination therapy with azacitidine and avelumab, another PD-L1 inhibitor, with a higher CRR of 10.5%, and two patients achieved CR with thrombocytopenia (Saxena et al., 2021). In addition to the aforementioned commonly used combination strategies, other epigenetic-immunotherapeutic approaches have also demonstrated promising efficacy. For instance, the combination of azacitidine with the CD47 monoclonal antibody, magrolimab, has shown notable clinical activity in patients with R/R AML, particularly those harboring TP53 mutations. In an enhanced-3 study, the median follow-up was 7.6 months (magrolimab arm) vs. 7.4 months (control arm), median OS was 10.7 vs. 14.1 months (Daver et al., 2025).

Moreover, epigenetic modulation has been shown to enhance the efficacy of monoclonal antibodies and bispecific T cell engagers (Masarova et al., 2017). Studies have demonstrated that DNMT inhibitors can upregulate the expression of target antigens such as CD33 or CD123 on the surface of AML cells, thereby increasing the binding affinity and cytotoxicity of antibody-based therapies (Zeng et al., 2024). A similar mechanism applies to improving the recognition efficiency of CAR-T cells in AML. Epigenetic drugs can increase target antigen density, attenuate T cell exhaustion phenotypes (e.g., by downregulating PD-1 and TIM-3 expression), and modulate the immunosuppressive bone marrow microenvironment, collectively enhancing CAR-T cell antileukemic activity (Saito and Nakazawa, 2024). Although the combination of epigenetic therapy and immunotherapy remains in the early stages of clinical investigation, with some patients experiencing immune-related adverse events and heterogeneous responses, this strategy has demonstrated promising potential. In the future, optimizing treatment timing, identifying predictive biomarkers of immune response, and integrating multi-omics data to enable precise immunomodulation are expected to become important directions in the development of AML therapy.

#### 3.2.4 Combination of multiple epigenetic drugs to overcome multi-mechanism resistance

In recent years, various therapeutic agents, including HDACi and DOT1L inhibitors, have demonstrated promising treatment potential for hematologic malignancies. However, extensive studies have revealed that monotherapy with these agents yields suboptimal clinical outcomes, whereas their combination with other drugs significantly improves patient response rates. Recent advances in epigenetic therapy have highlighted the therapeutic potential of combining agents that target distinct but interrelated layers of epigenetic regulation in AML. DNMT inhibitors, such as azacitidine, exert their anti-leukemic effects by reversing aberrant DNA methylation and reactivating silenced tumor suppressor and pro-differentiation genes. Besides, HDAC inhibitors and BETis act at the level of chromatin architecture, enhancing histone acetylation and increasing transcriptional accessibility of key regulatory pathways (Roboz et al., 2021; Garcia-Manero et al., 2024b). The mechanistic complementarity of these epigenetic modulators enables a more profound and coordinated transcriptional reprogramming than monotherapy alone. This combinatorial approach has been shown to effectively disrupt maintenance programs of LSCs, reverse epigenetic silencing of immune and apoptotic pathways, and reduce cellular plasticity that underlies resistance to conventional therapies. HDACi exhibit significant therapeutic potential in hematologic malignancies. Clinically approved HDACi agents, including chidamide and vorinostat, are currently utilized in AML treatment (San Jose-Eneriz et al., 2019). Chidamide, a selective HDAC1/2/3/10 inhibitor, induces oxidative stress-mediated DNA damage and demonstrates efficacy in R/R AML, which has been approved by the National Medical Products Administration (NMPA) in China (Wang et al., 2020). A phase I/II trial combining chidamide, DAC, aclarubicin, cytarabine, and G-CSF achieved 46% ORR (24 CR, 19 CRi) in 93 R/R AML patients, with responders showing prolonged OS (Wang et al., 2020). Vorinostat combined with DAC and cytarabine yielded 35% ORR in R/R AML with acceptable tolerability (Mims et al., 2018). DOT1L, a histone lysine methyltransferase targeting H3K79, catalyzes H3K79 methylation to drive leukemogenesis in MLL-rearranged (MLL-r) leukemias. EPZ-5676, a small-molecule DOT1L inhibitor targeting H3K79 methylation, demonstrated clinical activity in MLL-rearranged (MLL-r) AML. A multicenter dose-escalation study involving 51 R/R AML patients (37 KMT2Ar) revealed good tolerability (Grieselhuber and Mims, 2021). EPZ-5676 downregulates HOXA9/PBX3 expression and induces apoptosis in NPM1-mutated leukemia cells, establishing its therapeutic potential for MLL-r leukemias (Stein et al., 2021). A phase II clinical trial has shown that the combination of azacitidine and vorinostat in patients with R/R AML achieved an ORR rate of 71% and a CR rate of 35%. In contrast, treatment with azacitidine monotherapy demonstrated a lower ORR of 58% and a CR rate of only 13%, indicating a 13% increase in ORR with the combination regimen (Sanaei et al., 2018; Goh et al., 2020). The combination of azacitidine with spindolin exhibited promising results in a phase I clinical study, achieving an ORR of 43%, with 15% of patients attaining CR (Bewersdorf et al., 2019).

More recent preclinical studies suggest that triple epigenetic therapy, combining agents with distinct and complementary mechanisms, may provide a novel strategy to overcome drug resistance in AML. For instance, the combination of decitabine, panobinostat, and JQ1 has been proposed to target aberrant chromatin architecture and transcriptional dysregulation in AML cells. Although no clinical trials have yet evaluated this specific triplet, individual components and dual combinations have shown promising results in R/R AML models (Filippakopoulos et al., 2010; DeAngelo et al., 2019). This triple regimen holds potential to simultaneously modulate DNA methylation, histone acetylation, and oncogenic transcription factor expression, thereby reprogramming leukemic cells and enhancing therapeutic sensitivity. Future clinical investigations are warranted to validate the efficacy and safety of such multilayered epigenetic targeting strategie

## 4 Opportunities and challenges

Although conventional chemotherapy has improved remission rates to some extent, relapse and drug resistance remain major obstacles to successful treatment. The development of epigenetic therapies for AML, a highly heterogeneous hematologic malignancy, presents both exciting opportunities and significant challenges that shape current research directions. On the opportunity front, epigenetic modifications offer a unique therapeutic advantage that is potentially in AML treatment by regulating DNA methylation, histone modifications, and chromatin remodeling. These agents effectively restore tumor suppressor gene expression or inhibit oncogenic pathways, thereby overcoming resistance. The development of resistance is associated with multiple factors, including genetic mutations, adaptive changes in the tumor microenvironment, and epigenetic dysregulation. Among these mechanisms, the plasticity of epigenetic modifications makes them attractive targets for research and therapeutic intervention. In recent years, small-molecule epigenetic modulators have shown unique potential in AML treatment by regulating DNA methylation, histone modifications, and chromatin remodeling. These agents effectively restore tumor suppressor gene expression or inhibit oncogenic pathways, thereby overcoming resistance. Recent studies have demonstrated that DNMT inhibitors like azacitidine and decitabine can enhance tumor antigen presentation and potentially sensitize AML cells to immune checkpoint inhibitors, particularly in elderly AML patients who are ineligible for intensive chemotherapy, reopening new avenues for combination strategies (Chiappinelli et al., 2015; Skrbic et al., 2021). Beyond these established agents, novel compounds targeting histone modifications (including HDAC and BET inhibitors) and chromatin remodeling proteins (such as IDH1/2 inhibitors) are expanding the therapeutic landscape (Shafer and Grant, 2016). The emerging understanding of the immunomodulatory effects of epigenetic drugs represents another significant opportunity. Given the significant heterogeneity in the epigenetic landscape among AML patients, a single treatment approach may not be suitable for all cases. Advances in high-resolution epigenomic profiling technologies, including single-cell sequencing and chromatin accessibility assays, are enabling more precise patient stratification and the identification of predictive biomarkers for treatment response (Bandyopadhyay et al., 2024; Fiskus et al., 2024). Additionally, the development of more selective inhibitors and optimized dosing regimens could enhance patient selection and minimize adverse effects on normal cells. These approaches represent critical directions for future research.

Despite the promise of epigenetic-targeted therapy in AML, several challenges remain, including resistance mechanisms, lack of specificity, and the absence of predictive biomarkers. The inherent plasticity of AML cells enables rapid development of resistance through compensatory epigenetic reprogramming and activation of alternative survival pathways. This adaptive capacity is further complicated by the dynamic and heterogeneous nature of the AML epigenome, which varies both between patients and within individual patients over disease progression (Stelmach and Trumpp, 2023). The current lack of reliable predictive biomarkers makes it difficult to identify which patients are most likely to benefit from specific epigenetic therapies, while the broad mechanism of action of many epigenetic drugs contributes to off-target effects and toxicity concerns. Recent studies suggest that liquid biopsy-based epigenetic profiling may help address some of these monitoring challenges (Thakral et al., 2020). Additionally, the complex interplay between genetic and epigenetic alterations in AML requires more sophisticated preclinical models to better predict clinical responses (Chakraborty and Park, 2022). AML cells exhibit remarkable adaptability, and prolonged exposure to DNMT inhibitors may induce new epigenetic reprogramming, allowing tumor cells to proliferate via alternative signaling pathways. Moreover, compensatory gene expression changes can accelerate the emergence of resistance. Additionally, AML cells may bypass certain histone modifications through alternative epigenetic mechanisms. Furthermore, epigenetic-targeted drugs face challenges related to selectivity, side effects, and the lack of reliable predictive biomarkers. More combination therapies have emerged as a crucial strategy to address these issues. For instance, DNMT inhibitors combined with BET inhibitors or immune checkpoint inhibitors have demonstrated synergistic anti-leukemic effects, enhancing AML cell sensitivity to apoptotic signals while reducing resistance development (Du et al., 2021). In the future, an increasing number of dual therapies, and even triple or quadruple drug combinations, will be developed to address the issue of drug resistance in AML. Ultimately, the development of highly selective epigenetic inhibitors and rationally designed multi-drug regimens will be essential to address these obstacles, paving the way for improved clinical outcomes in AML.

## 5 Conclusion and perspectives

This review summarizes the role of epigenetic mechanisms in AML resistance, the research progress of small-molecule epigenetic-targeting drugs, and currently common combination therapies in clinical practice. Epigenetic regulation plays a crucial role in the initiation and progression of AML, and its plasticity makes it a key target for overcoming resistance. Strategies targeting epigenetic modifications have shown promising clinical potential, as they can regulate gene expression, inhibit tumor progression, and provide new therapeutic approaches to combat resistance. Although epigenetic-modulating drugs offer new possibilities for AML treatment, their clinical application still faces several challenges, including the development of resistance, issues with treatment specificity, side effects, and the lack of effective predictive biomarkers. Additionally, the highly heterogeneous epigenetic landscape among AML patients means that a single therapeutic approach may not suffice for all patients. Therefore, the development of more targeted and personalized treatment regimens is of utmost importance. To facilitate the clinical translation of epigenetic treatment strategies, this study proposes prioritizing two highly feasible initiatives based on existing technologies and platforms. On one hand, prospective biomarker-enriched cohorts could be established, focusing particularly on remethylation responders within DNMT3A-mutated acute myeloid leukemia. By leveraging the framework and biospecimen resources of large registry studies such as NCT03151408, in-depth characterization of molecular response features and clinical outcomes in this subpopulation can be achieved. On the other hand, it is advisable to promote MRD-driven dynamic treatment adjustment—specifically, within hypomethylating agent plus venetoclax regimens—where therapy intensity is individualized (escalated or de-escalated) based on serial MRD quantification (via multiparameter flow cytometry or sequencing). This strategy can be further validated and standardized through extension studies of phase III clinical trials such as VIALE-A. These two directions not only rest on solid clinical infrastructure but also hold strong potential to rapidly advance AML therapy from conventional regimens toward a biomarker-driven precision paradigm. Furthermore, emerging research is now turning a critical eye toward understanding how AML evolves epigenetically at relapse. Convergent epigenetic evolution—where relapsed AML cells adopt similar chromatin accessibility profiles irrespective of genetic stability—has been observed using single-cell ATAC-seq combined with mitochondrial tracing to map clonal trajectories from diagnosis to relapse (Nuno et al., 2024). Alongside this, single-cell epigenomics offers a powerful tool for dissecting the heterogeneity and uncovering resistance vulnerabilities at the individual-cell level—potentially illuminating new biomarkers and therapeutic entry points (Liu et al., 2024). Together, these lines of investigation underscore the importance of incorporating fine-resolution, cell-level insights and evolutionary dynamics to develop more nuanced, adaptive epigenetic treatment strategies in AML. These efforts aim to enhance therapeutic efficacy, reduce resistance development, and improve the long-term survival rates of patients. With advancements in technology and a deeper understanding of AML resistance mechanisms, breakthroughs in epigenetics hold the potential to provide more precise and effective treatment options for AML patients.

## Glossary

AML: Acute myeloid leukemia
OS: Overall survival
CR: Complete remission
LSCs: Leukemic stem cells
DNMT3A: DNA (cytosine-5)-methyltransferase 3 alpha
TET2: Ten-eleven translocation 2
IDH1/2: Isocitrate dehydrogenase 1/2
EZH2: Enhancer of zeste homolog 2
DNMTs: DNA methyltransferases
HDACs: Histone deacetylases
5mC: 5-methylcytosine
**5hm**C: 5-hydroxymethylcytosine
HMAs: hypomethylating agents
TCA: Tricarboxylic acid
α-KG: α-ketoglutarate
2-HG: 2-hydroxyglutarate
ABC: ATP-binding cassette
HATs: Histone acetyltransferases
H3K4me3: Trimethylation of H3K4
JMJD3: Jumanji Domain-Containing Protein 3
JMJD1A: Jumanji Domain-Containing Protein 1A
LSD1: Lysine-Specific Demethylase 1
MAPK: Mitogen-Activated Protein Kinase
H3S10: Histone H3 at serine 10
RNF2: RING Finger Protein 2
H2AK119ub: Histone H2A at lysine 119
ncRNAs: Non-coding RNAs
miRNAs: MicroRNAs
lncRNAs: Long non-coding RNAs
circRNAs: Circular RNAs
UHRF1: Ubiquitin-like with PHD and RING finger domains 1
CRR: Complete remission rates
HRQoL: Health-related quality of life
HCT: Hematopoietic cell transplantation
ICIs: Immune checkpoint inhibitors
MHC: Major histocompatibility complex
